# The association between the baseline bone resorption marker CTX and incident dysglycemia after 4 years

**DOI:** 10.1038/boneres.2017.20

**Published:** 2017-07-04

**Authors:** Ting-ting Liu, Dong-mei Liu, Yan Xuan, Lin Zhao, Li-hao Sun, Dian-dian Zhao, Xiao-feng Wang, Yang He, Xing-Zhi Guo, Rui Du, Ji-qiu Wang, Jian-min Liu, Hong-yan Zhao, Bei Tao

**Affiliations:** 1 Department of Endocrine and Metabolic Diseases, Ruijin Hospital, Shanghai Jiao Tong University School of Medicine; Shanghai Institute of Endocrine and Metabolic Diseases, Shanghai Clinical Center for Endocrine and Metabolic Diseases, Shanghai, China; 2 Department of Geriatrics, Ruijin Hospital North, Shanghai Jiao Tong University School of Medicine, Shanghai, China; 3 Department of Endocrine and Metabolic Diseases, Zhong Shan Hospital, Fudan University, Shanghai, China

## Abstract

Bone is an endocrine organ involved in modulating glucose homeostasis. The role of the bone formation marker osteocalcin (OCN) in predicting diabetes was reported, but with conflicting results. No study has explored the association between baseline bone resorption activity and incident diabetes or prediabetes during follow-up. Our objective was to examine the relationship between the baseline bone resorption marker crosslinked C-telopeptide of type I collagen (CTX) and glycemic dysregulation after 4 years. This longitudinal study was conducted in a university teaching hospital. A total of 195 normal glucose tolerant (NGT) women at baseline were invited for follow-up. The incidence of diabetes and prediabetes (collectively defined as dysglycemia) was recorded. A total of 128 individuals completed the 4-year study. The overall conversion rate from NGT to dysglycemia was 31.3%. The incidence of dysglycemia was lowest in the middle tertile [16.3% (95% confidence interval (CI), 6.8%–30.7%)] compared with the lower [31.0% (95% CI, 17.2%–46.1%)] and upper [46.5% (95% CI, 31.2%–62.6%)] tertiles of CTX, with a significant difference seen between the middle and upper tertiles (*P*=0.002 5). After adjusting for multiple confounding variables, the upper tertile of baseline CTX was associated with an increased risk of incident dysglycemia, with an odds ratio of 7.09 (95% CI, 1.73–28.99) when the middle tertile was the reference. Osteoclasts actively regulate glucose homeostasis in a biphasic model that moderately enhanced bone resorption marker CTX at baseline provides protective effects against the deterioration of glucose metabolism, whereas an overactive osteoclastic function contributes to an increased risk of subsequent dysglycemia.

## Introduction

Diabetes is now a worldwide health problem.^1^ Epidemiological studies have also reported a prevalence of prediabetes as high as 30%–50%.^2,3^ It was estimated that 20%–25% of normal glucose tolerant (NGT) subjects will develop prediabetes and diabetes in 10 years;^4^ thus, there is an urgent need to identify subjects at high risk of developing prediabetes and diabetes.

Bone is an ever-changing organ that actively participates in the regulation of energy homeostasis.^5^ In the last 10 years, mounting evidence from mouse models has suggested a promising role of the skeleton in regulating glucose homeostasis, mainly through a bone protein synthesized by osteoblasts called osteocalcin (OCN), which can stimulate insulin release from β cells and improve insulin sensitivity.^6,7,8,9^ Although most, but not all, human cross-sectional investigations lend support to the negative associations between the bone formation marker OCN and fasting plasma glucose (FPG) and glycated hemoglobin (HbA1c),^10^ data from a limited number of longitudinal human studies are not consistent.^11,12^

The underlying reason for these controversial clinical observations is multi-factorial. Although most studies focused on OCN produced by osteoblasts, the function of osteoclasts in modulating energy metabolism should not be neglected. A mouse study demonstrated that changes in osteoclast number or activity directly altered glucose tolerance.^13^ It was also shown that osteoclasts promote OCN bioactivity by inducing its decarboxylation in the acidic resorptive environment that they create.^14^ Recently, there is important and novel evidence emerging that indirectly suggests a link between osteoclast activity and energy metabolism.^15,16^ Clinically, in our previous cross-sectional study, we found that in NGT subjects, HbA1c is a positive contributor to the changes in the bone resorption marker crosslinked C-telopeptide of type I collagen (CTX), and that the increased serum concentrations of CTX were negatively associated with homoeostasis model assessment for insulin resistance.^17^ These findings suggested that in NGT subjects, when there is a trend of increasing HbA1c, CTX will increase to counterbalance the continuing deterioration of glucose homeostasis. We further noted the protective compensatory response of bone peaks in the prediabetes status. It was thus hypothesized that with time, the skeleton may become “exhausted,” leading to the development of diabetes.^17^ On the basis of this assumption, it is necessary to examine whether there is really an “optimal” range for the glucose-protective effects of osteoclastic bone resorption and to determine under what conditions the osteoclasts will become “exhausted” and confer an increased risk of incident diabetes.

Thus, to verify our bone compensation/exhaustion model in the occurrence of glycemic dysregulation, we followed the NGT subjects from our previous study for 4 years and found that slightly enhanced osteoclast activity, as reflected by the middle tertile of baseline CTX, is associated with the lowest risk of conversion from NGT to prediabetes and type 2 diabetes (collectively defined as dysglycemia). An exaggerated bone resorption function at baseline, as defined by the upper tertile of CTX, is predictive of developing dysglycemia during follow-up.

## Materials and methods

In our initial cross-sectional study, there were 195 NGT women whose condition was confirmed by a 75-g oral glucose tolerance test,^17^ and all of them were invited to participate in this 4-year follow-up study. Among these individuals, 45 migrated to other cities, 21 were reluctant to participate, and 1 died of pancreatic carcinoma; thus, 128 subjects (65.6%) were investigated in the current study. Serum concentrations of OCN and CTX were measured as previously reported.^17^ Serum levels of 25-OH-D were measured by automatic electrochemiluminescence immunoassay (Cobas e602, Roche Diagnostics GmbH, Mannheim, Germany).

During the follow-up visit, oral glucose tolerance test was again performed after a 10-h fast in all subjects. FPG and 2-h plasma glucose (2-hPG) levels were measured during the oral glucose tolerance test. Anthropometric parameters, including age, body height, body weight, waist circumference, hip circumference, and blood pressure, were recorded as described previously.^17^ No one was taking active anti-resorptive medications, including bisphophonates, hormone replacement therapy, selective estrogen receptor modulators, or parathyroid hormone (1–34) at baseline. All participants provided written informed consent. The Ethics Committee of Ruijin Hospital, Shanghai Jiao Tong University School of Medicine approved the study.

### Outcome assessment

Diabetes was diagnosed if 2-hPG was ≥11.1 mmol·L^−1^ and/or the FPG level was ≥7.0 mmol·L^−1^. History of diabetes was obtained through self-report at follow-up. Prediabetes (impaired fasting glucose/impaired glucose tolerance) was defined as FPG 6.1–6.9 mmol·L^−1^ and/or 2-hPG 7.8–11.0 mmol·L^−1^. Dysglycemia was defined as individuals having a diabetic or prediabetic state. NGT individuals were those with FPG <6.1 mmol·L^−1^ and 2-hPG <7.8 mmol·L^−1^.

### Statistical analyses

Statistical analyses were performed using SAS Statistical Package (version 9.0; SAS Institute, Cary, NC, USA). Participants’ characteristics were represented using the mean±s.d. for continuous variables and medians with 25th and 75th percentiles for abnormally distributed variables. Differences between groups were analyzed using Student’s *t*-test or an analysis of variance, and log transformation was applied where appropriate. Wilcoxon signed rank-sum tests or Kruskal–Wallis tests were performed for nonparametric statistics. Categorical variables were shown as counts and proportions, and the differences were analyzed using Pearson *χ*^2^-tests. The odds ratio (OR) and 95% confidence interval (CI) of the association were assessed by logistic regression analysis. A *P*-value <0.05 was considered statistically significant.

## Results

The baseline characteristics of the participants according to CTX tertiles are shown in Table 1. Stepwise increases in age and the proportion of post-menopausal women were observed with increasing CTX tertiles (both *P*<0.000 1). No differences were observed in terms of body mass index or waist-to-hip ratio across CTX tertiles. Participants in the upper tertile had significantly higher levels of HbA1c than those in the middle (*P*<0.01) and lower tertiles (*P*<0.05). However, there were no differences in FPG, 2-hPG, fasting insulin, or homoeostasis model assessment for insulin resistance among the CTX tertiles. Serum concentrations of OCN (*P*<0.000 1), calcium (*P*=0.007 5), and albumin-adjusted calcium (*P*=0.002 8) increased with CTX tertiles, whereas bone mineral densities (BMDs) at lumbar spine L1–4 (*P*<0.000 1), femoral neck (*P*=0.000 8), and total hip (*P*=0.000 2) showed decreasing tendencies. There was no significant difference in serum 25-OH-D levels across CTX tertiles. CTX tertiles were not associated with hypertension, lipid profile, or renal function.

### Changes of glycemic status during follow-up

Over a median of 4.0 (range, 3.2–4.2) years follow-up, 4 (3.1%) out of 128 individuals developed diabetes and 36 (28.1%) progressed from NGT to prediabetes, giving an overall conversion rate to dysglycemia of 31.3%. Levels of FPG, 2-hPG, and HbA1c at the end of follow-up increased significantly compared with those at baseline (FPG, 5.3 (5.1–5.6) vs 5.5 (5.1–5.7) mmol·L^−1^, *P*=0.000 2; 2-hPG, 5.9 (5.3–6.6) vs 6.9 (6.0–7.9) mmol·L^−1^, *P*<0.000 1; HbA1c, 5.5 (5.3–5.7) % vs 5.6 (5.4–5.7) %, *P*=0.011 9).

### Incident dysglycemia and baseline CTX levels

**Figure 1:**
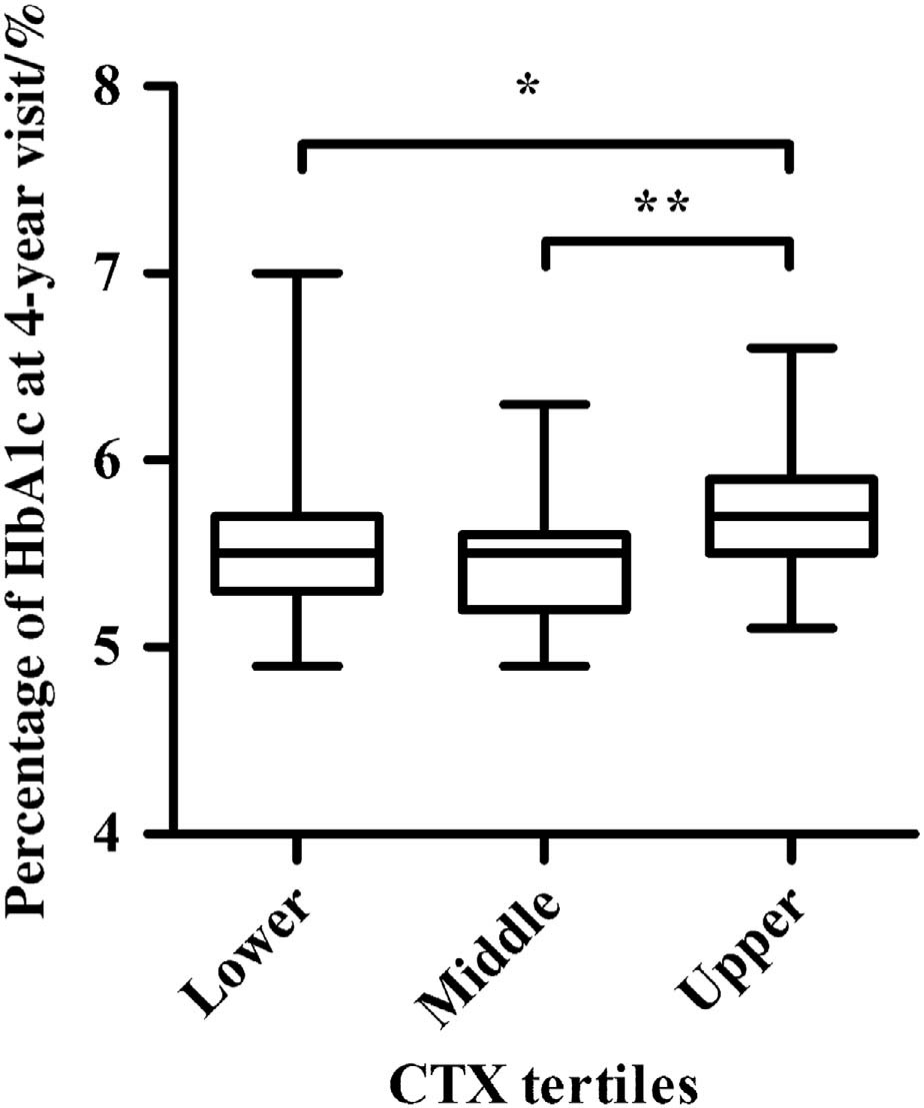
HbA1c at the follow-up according to baseline CTX tertiles. Data are shown as the mean (minimum, maximum). *P<0.05, **P<0.01.

**Figure 2:**
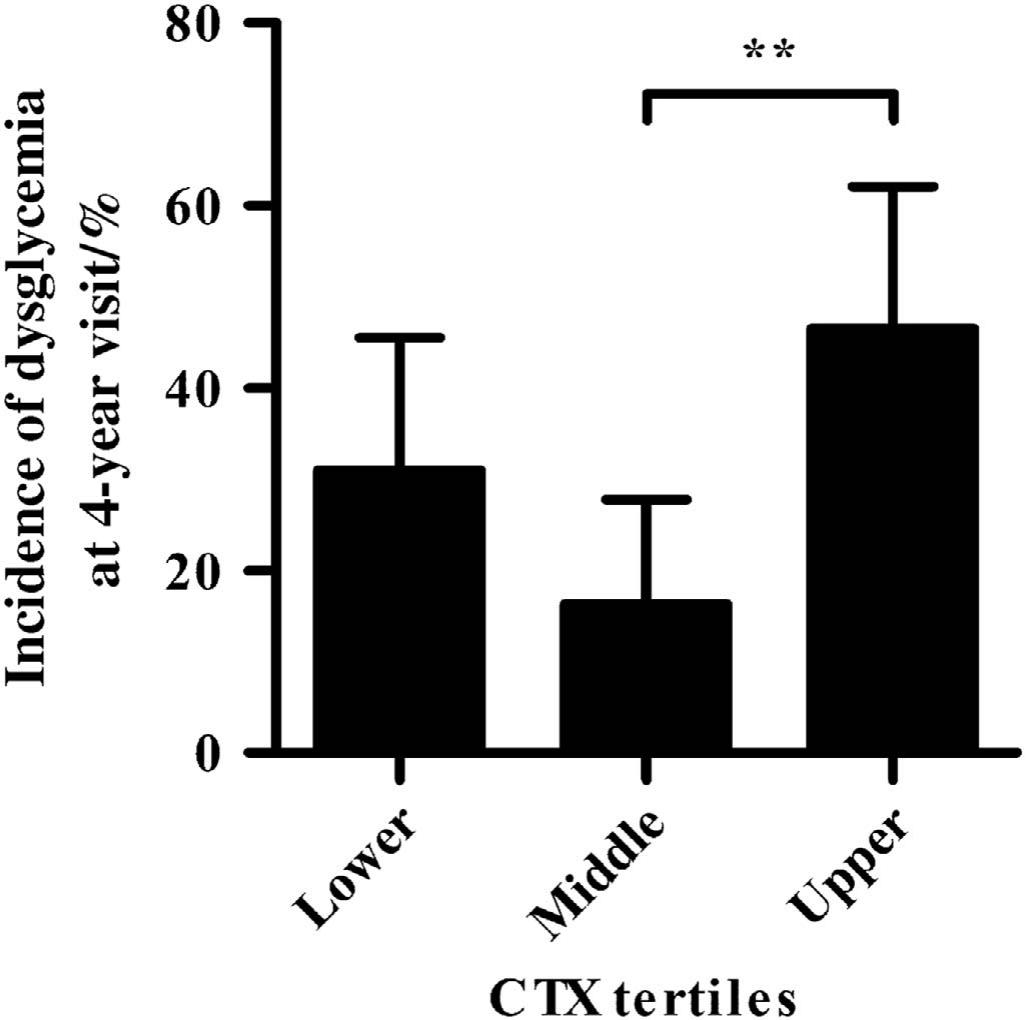
Incidence of dysglycemia at the follow-up according to baseline CTX tertiles. Data are shown as the mean (95% CIs). **P<0.01.

During follow-up, the 2-hPG (Table 2) and HbA1c (Figure 1) values of individuals in the upper baseline CTX tertile were higher than those in the middle (both *P*<0.01) and lower tertiles (both *P*<0.05). Individuals in the upper baseline CTX tertile had elevated FPG compared with those in the middle tertile (*P*<0.05). The incidence of type 2 diabetes was 4.7% in the upper tertile, 2.3% in the middle tertile, and 2.4% in the lower tertile, but the differences were not significant. The incidence of prediabetes was lowest in the middle tertile (14.0%), which was lower than those of the upper tertile (41.9%, *P*=0.003 9) and lower tertile of CTX (28.6%, *P*=0.099 1). As shown in Figure 2, the incidence rates of dysglycemia were 31.0% (95% CI, 17.2%–46.1%), 16.3% (95% CI, 6.8%–30.7%), and 46.5% (95% CI, 31.2%–62.6%) in the three tertiles, with a significant difference between the middle and upper tertiles (*P*=0.002 5).

We then computed the ORs and 95% CIs for incident dysglycemia according to baseline CTX tertiles with and without the adjustment of several confounders. Because the incidence of dysglycemia was lowest in the middle CTX tertile, this tertile was chosen as the reference. As shown in Table 3, subjects in the upper and lower tertiles of CTX had an increased risk of incident dysglycemia compared with the reference tertile, and statistical significance was evident for the upper tertile [OR, 4.47(95% CI, 1.63–12.25)]. Such an association still existed and even improved when age, body mass index, menopause status, hypertension, triglyceride, high-density lipoprotein, low-density lipoprotein, total cholesterol, uric acid, urea nitrogen, and creatinine levels were adjusted for, and the OR of dysglycemia in the upper CTX tertile was 4.98 (95% CI, 1.46–17.03). When bone mineral densities at L1–4, femoral neck, and total hip were further adjusted, the OR of dysglycemia reached 6.25 (95% CI, 1.67–23.43). When we additionally adjusted albumin-adjusted calcium and OCN, the upper tertile of CTX was still significantly associated with an increased risk of incident dysglycemia, with a higher OR of 7.09 (95% CI, 1.73–28.99).

## Discussion

The most important finding of the current study, which was performed in a group of NGT individuals, is that moderately enhanced bone resorptive activity at baseline, that is, the middle tertile of bone resorption marker CTX, contributes to a lower risk of progression from NGT to dysglycemia in 4 years, whereas overactive osteoclast function is predictive of the subsequent development of prediabetes and diabetes, independent of OCN.

Previous mouse studies clearly indicated that the formation of the metabolically active form of OCN, which can augment insulin release from pancreatic β cells and improve insulin resistance, relies on the acidic bone microenvironment provided by enhanced osteoclastic activity.^14,18,19^ In addition, the glucose-regulatory effects of osteoclasts were further demonstrated in mouse models with loss or gain of function of bone resorption.^13^ It was revealed that ablation of osteoclasts prenatally or post-developmentally results in the deterioration of glucose tolerance and impairment of insulin secretion due to decreased β-cell mass and islet number.^13^ By contrast, mice with increased bone resorption as a result of deleting osteoprotegerin, a critical suppressive cytokine of osteoclastic activities, are more glucose tolerant and insulin sensitive than wild-type mice.^13^

These findings from mouse studies prompted us to explore the implication of osteoclasts in glucose homeostasis in humans. In our previous cross-sectional study, after a detailed analysis of the relationships between CTX and HbA1c, we assumed that the elevation of CTX in prediabetes may represent a compensatory response of bone to protect against the mild deterioration of glucose homeostasis.^17^ This assumption was tested and confirmed in this follow-up study. We found that a slightly enhanced osteoclast activity at baseline, for example, the middle tertile of CTX at 0.29 (0.25–0.33) ng·mL^−1^, conferred the lowest risk of developing dysglycemia after 4 years. Although not unanimously consistent, it has already been demonstrated that bone formation and resorption markers in type 2 diabetic patients were lower in concentration than those in normal controls.^20,21^ Meanwhile, in a large-scale cross-sectional study conducted in older men, it was found that for every 1 s.d. increase in bone formation and resorption parameters, including OCN and its undercarboxylated form, CTX, and N-terminal propeptide of type I collagen, there was a 30%–50% reduction in diabetes risk.^22^ This finding, together with ours, suggests that somewhat rapid bone remodeling might be necessary for preventing the progression of NGT to prediabetes and diabetes.

However, this compensatory effect may ultimately be exhausted.^17^ In this study, we noted that those NGT subjects already at the upper tertile of baseline serum CTX concentrations [0.47 (0.41–0.56) ng·mL^−1^] had an increased risk of subsequent dysglycemia, even after adjusting for multiple confounders, including age, body mass index, hypertension, medications, lipid profile, and others. Similar to us, a prospective population-based study reported that individuals who are in the upper tertile of serum concentration of receptor activator for nuclear factor-κ B ligand, which is a potent stimulator of nuclear factor-κB and a crucial modulator of bone resorption,^23,24^ have a three- to four-fold increase in the risk of developing type 2 diabetes compared with those in the median and low tertiles.^25^ An increased serum calcium level, which could be regarded as a result of high bone turnover, was shown to be a predictor of type 2 diabetes.^26^ By contrast, anti-resorptive therapy in a mouse model of type 2 diabetes improved insulin sensitivity and ameliorated or even normalized plasma glucose concentrations and glucose tolerance.^25^ In addition, in humans, a 30%–50% or greater risk reduction for the development of type 2 diabetes was shown in users of anti-resorptive drugs compared with nonusers.^27,28^ Although the serum concentrations of bone turnover markers after treatment were not described in these studies, we tried to explain all these findings by a biphasic model of bone resorption in modulating glucose homeostasis .In our model, a moderately increased bone resorption activity is beneficial to glucose metabolism, whereas over-exaggerated bone resorption activity is harmful. According to our results, it could be inferred that preserving bone resorption within a certain range, for example, a middle tertile of serum CTX level at ~0.25–0.30 ng·mL^−1^, might provide protection against the dysregulation of glucose metabolism. Meanwhile, enhanced bone resorption activity with CTX serum concentration greater than 0.47 (0.41–0.56) ng·mL^−1^ confers the greatest risk of dysglycemia. If proven to be correct in larger clinical studies, our results have important clinical implications, especially when osteoporotic post-menopausal women are receiving anti-resorptive therapies: high bone turnover should be inhibited, but not too much, for the sake of preventing diabetes.

Previous results from rodent and human studies have suggested that the glucose effects of CTX are mediated through OCN.^13,17^ In the current study, even with the adjustment of OCN, the middle tertile CTX is still associated with the lowest risk of subsequent dysglycemia. This result suggests that risk reduction of dysglycemia with respect to a moderately increased CTX is independent of OCN, thus confirming that other molecule(s) might be involved in the interactions between bone and energy metabolism.^29^ In addition, it was recently shown that with the global or selective ablation of leucine-rich-repeat-containing G protein-coupled receptor 4 (LGR4) in monocytes, which are the precursors of osteoclasts, the mutant mice not only developed osteoporosis with an increased osteoclast number and size^15^ but were also resistant to diet and leptin-mutant-induced obesity with improved glucose metabolism.^16^ Furthermore, a variety of phenotypes, including low bone mass, osteoporotic fracture, lower body weight and so on, were reported in Icelandic subjects with a rare loss-of-function mutation in LGR4.^30^ Thus, it is of interest to investigate whether the glucose effects of CTX might be mediated through LGR4 and its related signaling pathway.

Vitamin D status may be a confounding factor implicated in the cross-talk between bone and glucose metabolism, as a lower serum 25-OH-D level has been regarded as a risk factor for diabetes.^31,32^ Meanwhile, in our study, serum levels of 25-OH-D were comparable in the three groups of CTX tertiles. Despite the small sample size of the current study, several long-term clinical studies also reported that supplementation of vitamin D, even with very high dosages, could not reduce the risk of type 2 diabetes in prediabetes with or without hypovitaminosis D.^33,34^

CTX was recommended as a classical bone remodeling marker.^35^ The possible effects of exposure to hyperglycemia on the measurement of CTX should be considered .Collagens in the body could be glycated by non-enzymatic reactions,^36^ and the formation of advanced glycated end products is involved in the pathogenesis of diabetes and its chronic complications.^37,38^ The glycation of bone collagen may interfere with osteoclast differentiation and activity,^39^ compromise bone quality and be responsible for the higher fracture risk in type 2 diabetic patients.^37,40,41^ As CTX is the product of type I collagen, there is the possibility that the degraded type I collagen might have already been glycated, especially in diabetic patients. However, to our knowledge, there is no such method that can differentiate glycated CTX from unglycated CTX. Further studies are needed to explore the extent to which the non-enzymatic glycation of bone collagen could influence bone resorption activity and its peripheral measurements.

There are several limitations of this study. (1) The sample size is small. The conversion rate from NGT to prediabetes in this study was 28.1%, a 25.7% conversion rate was reported in an Asian Indian 10-year follow-up study.^4^ However, the progression from NGT to diabetes was much lower in this study (3.1%) than in others (7.3%–19.3%).^4,12,26,42^ The low follow-up rate might be a cause for this discrepancy. (2) Only one bone resorption marker, CTX, was measured and no data regarding other bone resorption parameters, such as tartrate-resistant acid phosphatase 5b, were shown. Tartrate-resistant acid phosphatase 5b is secreted by osteoclasts, but its instability^43,44^ and tendency to method-related systemic error^45^ limits its wide use. Recently, higher bone remodeling, rather than a specific bone turnover marker, was reported to be predictive of a lower incidence of diabetes.^22^ A comprehensive evaluation of bone remodeling status with a panel of bone biomarkers should still be stressed.

In conclusion, we summarize that the correlation between serum CTX and incident dysglycemia is probably a U-shaped graph, and the same can be observed for the relationships between all-cause mortality and serum 25(OH) D,^46^ OCN,^47^ and HbA1c.^48^ Overall, this follow-up study further strengthens the hypothesis developed after our initial cross-sectional study,^17^ which can be summarized as follows: (1) during the early stages of glucose dysregulation, even in the NGT stage, bone may react by increasing osteoclast activity to improve glucose homeostasis; (2) an overactive bone resorption process may exhaust bone, rendering it unable to provide further protective effects against the deterioration of glucose homeostasis; and (3) maintaining bone remodeling at an optimal range, as shown in this study within the middle tertile of CTX, is beneficial for preventing the transition from NGT to prediabetes and diabetes. Clearly, more evidence is required to verify the involvement of osteoclasts as active bone endocrine cells in regulating glucose metabolism.

## Figures and Tables

**Table 1:** Baseline characteristics according to CTX tertiles

	Tertiles of CTX			
Baseline characteristics	Lower (*n*=42)	Middle (*n*=43)	Upper (*n*=43)	*P*-value
CTX/(ng·mL^−1^)	0.18 (0.12–0.21)	0.29 (0.25–0.33)	0.47 (0.41–0.56)	<0.000 1
Age/years	50 (40–60)	54 (45–57)	58 (56–64)	<0.000 1
BMI/(kg·m^−2^)	23.14(21.77–24.77)	22.49 (20.79–24.39)	22.17 (20.55–23.83)	0.214 8
Waist-to-hip ratio	0.85±0.06	0.82±0.05	0.83±0.06	0.110 5
Menopause/*n*	16 (38.1%)	23 (53.5%)	38 (88.4%)	<0.000 1
HbA1c/%	5.5 (5.2–5.7)	5.4 (5.2–5.6)	5.7 (5.4–6.0)	0.001 0
Insulin/(mIU·L^−1^)	7.06 (4.53–9.39)	6.94 (3.91–8.94)	5.99 (4.83–7.58)	0.160 9
FPG/(mmol·L^−1^)	5.2 (4.9–5.6)	5.3 (5.0–5.5)	5.3 (5.2–5.7)	0.210 6
2-hPG/(mmol·L^−1^)	6.1±0.9	5.7±0.9	6.0±1.0	0.110 1
HOMA IR	1.68 (1.08–2.30)	1.58 (1.03–2.15)	1.52 (1.05–1.78)	0.115 4
Hypertension/*n*	3 (7.5%)	8 (19.5%)	12 (27.9%)	0.056 4
Triglyceride/(mmol·L^−1^)	1.00 (0.76–1.59)	1.02 (0.82–1.53)	1.10 (0.86–1.65)	0.311 9
Total cholesterol/(mmol·L^−1^)	5.09 (4.58–6.05)	5.24 (4.53–6.04)	5.25 (4.75–5.79)	0.931 9
HDL/(mmol·L^−1^)	1.68±0.36	1.70±0.40	1.66±0.36	0.889 1
LDL/(mmol·L^−1^)	3.11 (2.60–3.80)	3.18 (2.48–3.60)	3.17 (2.68–3.65)	0.807 3
Uric acid/(μmol·L^−1^)	281 (254–304)	267 (221–316)	274 (245–309)	0.291 5
Urea nitrogen/(μmol·L^−1^)	4.77±1.05	4.65±0.97	4.69±0.95	0.855 8
Creatinine/(μmol·L^−1^)	54 (51–60)	56 (51–62)	57 (53–63)	0.229 8
OCN/(ng·mL^−1^)	12.96 (11.20–14.67)	17.14 (14.64–19.55)	23.08 (18.52–27.91)	<0.000 1
Ca/(mmol·L^−1^)	2.26±0.08	2.31±0.09	2.32±0.10	0.007 5
Albumin-adjusted calcium/(mmol·L^−1^)	2.22±0.08	2.25±0.09	2.28±0.08	0.002 8
25-OH-D/(nmol·L^−1^)^a^	32.99 (29.54–42.31)	36.48 (29.22–42.00)	40.63 (33.42–44.43)	0.733 4
L1–4 BMD/(g·cm^−2^)	1.18±0.20	1.11±0.18	0.97±0.15	<0.000 1
Femoral neck BMD/(g·cm^−2^)	0.90±0.15	0.89±0.11	0.80±0.10	0.000 8
Total hip BMD/(g·cm^−2^)	0.97±0.16	0.93±0.13	0.85±0.11	0.000 2

BMD, bone mineral density; BMI, body mass index; CTX, C-telopeptide of type I collagen; FPG, fasting plasma glucose; HOMA IR, homoeostasis model assessment for insulin resistance; HDL, high-density lipoprotein; LDL, low-density lipoprotein.

Data are presented as *n* (%), means±s.d. or medians (interquartile range); Hypertension was defined as systolic blood pressure ≥140 mm Hg or diastolic blood pressure ≥90 mm Hg or reporting antihypertensive medication use.

a Tested in 18, 15 and 15 subjects in the three groups, respectively.

**Table 2:** FPG, 2-hPG, and glycemic status at a 4-year follow-up according to baseline CTX tertiles

	Tertiles of CTX			
Parameters	Lower (*n*=42)	Middle (*n*=43)	Upper (*n*=43)	*P-*value
Duration of follow-up/years	4.0 (3.5–4.0)	4.0 (3.5–4.1)	4.0 (3.5–4.1)	0.584 3
FPG/(mmol·L^−1^)	5.4 (5.0–5.7)	5.4 (5.1–5.6)	5.7 (5.3–6.0)	0.030 9
2-hPG/(mmol·L^−1^)	6.6 (5.8–7.7)	6.7 (5.7–7.2)	7.4 (6.8–8.5)	0.007 6
Diabetes/*n*	1 (2.4%)	1 (2.3%)	2 (4.7%)	0.779 4
Prediabetes/*n*	12 (28.6%)	6 (14.0%)	18 (41.9%)	0.015 8
NGT/*n*	29 (69.1%)	36 (83.7%)	23 (53.5%)	0.010 3
Dysglycimia/*n*	13 (31%)	7 (16.3%)	20 (46.5%)	0.010 3

2-hPG, 2-h plasma glucose in OGTT; CTX, C-telopeptide of type I collagen; FPG, fasting plasma glucose; NGT, normal glucose tolerance; OGTT, oral glucose tolerance test.

**Table 3:** Odds ratio of incident dysglycemia according to baseline CTX tertiles

	Tertiles of CTX		
Parameters	Lower	Middle	Upper
Crude model	2.31 (0.81–6.53)	1 (ref.)	4.47 (1.63–12.25)
Model 1	2.20 (0.72–6.78)	1 (ref.)	4.49 (1.46–13.75)
Model 2	2.99 (0.86–10.43)	1 (ref.)	4.98 (1.46–17.03)
Model 3	2.85 (0.81–9.99)	1 (ref.)	6.25 (1.67–23.43)
Model 4	2.45 (0.64–9.41)	1 (ref.)	7.09 (1.73–28.99)

BMD, bone mineral density; BMI, body mass index; CI, confidence interval; CTX, C-telopeptide of type I collagen; HDL, high-density lipoprotein; LDL, low-density lipoprotein; OCN, osteocalcin; OR, odds ratio; ref., reference.

Results are expressed as ORs (95% CIs) for dysglycemia: model 1 was adjusted for age and BMI. Model 2 was additionally adjusted for menopause, hypertension, triglyceride, HDL, LDL, total cholesterol, uric acid, urea nitrogen, and creatinine. Model 3 was additionally adjusted for BMDs at L1–4, femoral neck, and total hip. Model 4 was additionally adjusted for albumin-corrected calcium and OCN.

## References

[bib1] Fishman EI, Stokes A, Preston SH. The dynamics of diabetes among birth cohorts in the U.S. Diabetes Care 2014; 37: 1052–1059. 24513590 10.2337/dc13-1982 PMC3964490

[bib2] Xu Y, Wang L, He J et al. Prevalence and control of diabetes in Chinese adults. JAMA 2013; 310: 948–959. 24002281 10.1001/jama.2013.168118

[bib3] Mota M, Popa SG, Mota E et al. Prevalence of diabetes mellitus and prediabetes in the adult Romanian population: PREDATORR study. J Diabetes 2016; 8: 336–344. 25850521 10.1111/1753-0407.12297

[bib4] Anjana RM, Shanthi Rani CS, Deepa M et al. Incidence of diabetes and prediabetes and predictors of progression among Asian Indians: 10-year follow-up of the Chennai Urban Rural Epidemiology Study (CURES). Diabetes Care 2015; 38: 1441–1448. 25906786 10.2337/dc14-2814

[bib5] Karsenty G, Oury F. Biology without walls: the novel endocrinology of bone. Annu Rev Physiol 2012; 74: 87–105. 22077214 10.1146/annurev-physiol-020911-153233 PMC9277655

[bib6] Lee NK, Sowa H, Hinoi E et al. Endocrine regulation of energy metabolism by the skeleton. Cell 2007; 130: 456–469. 17693256 10.1016/j.cell.2007.05.047 PMC2013746

[bib7] Wei J, Shimazu J, Makinistoglu MP et al. Glucose uptake and Runx2 synergize to orchestrate osteoblast differentiation and bone formation. Cell 2015; 161: 1576–1591. 26091038 10.1016/j.cell.2015.05.029 PMC4475280

[bib8] Ferron M, Hinoi E, Karsenty G et al. Osteocalcin differentially regulates beta cell and adipocyte gene expression and affects the development of metabolic diseases in wild-type mice. Proc Natl Acad Sci USA 2008; 105: 5266–5270. 18362359 10.1073/pnas.0711119105 PMC2278202

[bib9] Ferron M, McKee MD, Levine RL et al. Intermittent injections of osteocalcin improve glucose metabolism and prevent type 2 diabetes in mice. Bone 2012; 50: 568–575. 21550430 10.1016/j.bone.2011.04.017 PMC3181267

[bib10] Liu DM, Guo XZ, Tong HJ et al. Association between osteocalcin and glucose metabolism: a meta-analysis. Osteoporos Int 2015; 26: 2823–2833. 26089135 10.1007/s00198-015-3197-8

[bib11] Ngarmukos C, Chailurkit LO, Chanprasertyothin S et al. A reduced serum level of total osteocalcin in men predicts the development of diabetes in a long-term follow-up cohort. Clin Endocrinol 2012; 77: 42–46. 10.1111/j.1365-2265.2011.04215.x 21916911

[bib12] Hwang YC, Jee JH, Jeong IK et al. Circulating osteocalcin level is not associated with incident type 2 diabetes in middle-aged male subjects: mean 8.4-year retrospective follow-up study. Diabetes Care 2012; 35: 1919–1924. 22773701 10.2337/dc11-2471 PMC3424992

[bib13] Lacombe J, Karsenty G, Ferron M. *In vivo* analysis of the contribution of bone resorption to the control of glucose metabolism in mice. Mol Metab 2013; 2: 498–504. 24327965 10.1016/j.molmet.2013.08.004 PMC3854996

[bib14] Ferron M, Wei J, Yoshizawa T et al. Insulin signaling in osteoblasts integrates bone remodeling and energy metabolism. Cell 2010; 142: 296–308. 20655470 10.1016/j.cell.2010.06.003 PMC2910411

[bib15] Luo J, Yang Z, Ma Y et al. LGR4 is a receptor for RANKL and negatively regulates osteoclast differentiation and bone resorption. Nat Med 2016; 22: 539–546. 27064449 10.1038/nm.4076

[bib16] Wang J, Liu R, Wang F et al. Ablation of LGR4 promotes energy expenditure by driving white-to-brown fat switch. Nat Cell Biol 2013; 15: 1455–1463. 24212090 10.1038/ncb2867

[bib17] Xuan Y, Sun LH, Liu DM et al. Positive association between serum levels of bone resorption marker CTX and HbA1c in women with normal glucose tolerance. J Clin Endocrinol Metab 2015; 100: 274–281. 25343234 10.1210/jc.2014-2583

[bib18] Karsenty G, Ferron M. The contribution of bone to whole-organism physiology. Nature 2012; 481: 314–320. 22258610 10.1038/nature10763 PMC9047059

[bib19] Liu JM, Rosen CJ, Ducy P et al. Regulation of glucose handling by the skeleton: insights from mouse and human studies. Diabetes 2016; 65: 3225–3232. 27959858 10.2337/db16-0053 PMC5860442

[bib20] Yamamoto M, Yamaguchi T, Nawata K et al. Decreased PTH levels accompanied by low bone formation are associated with vertebral fractures in postmenopausal women with type 2 diabetes. J Clin Endocrinol Metab 2012; 97: 1277–1284. 22337915 10.1210/jc.2011-2537

[bib21] Manavalan JS, Cremers S, Dempster DW et al. Circulating osteogenic precursor cells in type 2 diabetes mellitus. J Clin Endocrinol Metab 2012; 97: 3240–3250. 22740707 10.1210/jc.2012-1546 PMC3431571

[bib22] Yeap BB, Alfonso H, Chubb SA et al. Higher serum undercarboxylated osteocalcin and other bone turnover markers are associated with reduced diabetes risk and lower estradiol concentrations in older men. J Clin Endocrinol Metab 2015; 100: 63–71. 25365314 10.1210/jc.2014-3019

[bib23] Lacey DL, Timms E, Tan HL et al. Osteoprotegerin ligand is a cytokine that regulates osteoclast differentiation and activation. Cell 1998; 93: 165–176. 9568710 10.1016/s0092-8674(00)81569-x

[bib24] Fata JE, Kong YY, Li J et al. The osteoclast differentiation factor osteoprotegerin-ligand is essential for mammary gland development. Cell 2000; 103: 41–50. 11051546 10.1016/s0092-8674(00)00103-3

[bib25] Kiechl S, Wittmann J, Giaccari A et al. Blockade of receptor activator of nuclear factor-kappaB (RANKL) signaling improves hepatic insulin resistance and prevents development of diabetes mellitus. Nat Med 2013; 19: 358–363. 23396210 10.1038/nm.3084

[bib26] Becerra-Tomás N, Estruch R, Bulló M et al. Increased serum calcium levels and risk of type 2 diabetes in individuals at high cardiovascularrisk. Diabetes Care 2014; 37: 3084–3091. 25139884 10.2337/dc14-0898

[bib27] Toulis KA, Nirantharakumar K, Ryan R et al. Bisphosphonates and glucose homeostasis: a population-based, retrospective cohort study. J Clin Endocrinol Metab 2015; 100: 1933–1940. 25695881 10.1210/jc.2014-3481

[bib28] Vestergaard P. Risk of newly diagnosed type 2 diabetes is reduced in users of alendronate. Calcif Tissue Int 2011; 89: 265–270. 21710315 10.1007/s00223-011-9515-z

[bib29] Yoshikawa Y, Kode A, Xu L et al. Genetic evidence points to an osteocalcin-independent influence of osteoblasts on energy metabolism. J Bone Miner Res 2011; 26: 2012–2025. 21557308 10.1002/jbmr.417 PMC3656486

[bib30] Styrkarsdottir U, Thorleifsson G, Sulem P et al. Nonsense mutation in the LGR4 gene is associated with several human diseases and other traits. Nature 2013; 497: 517–520. 23644456 10.1038/nature12124

[bib31] Veronese N, Sergi G, De Rui M et al. Serum 25-hydroxyvitamin D and incidence of diabetes in elderly people: the PRO.V.A. study. J Clin Endocrinol Metab 2014; 99: 2351–2358. 24731010 10.1210/jc.2013-3883

[bib32] Xuan Y, Zhao HY, Liu JM. Vitamin D and type 2 diabetes mellitus (D2). J Diabetes 2013; 5: 261–267. 23302127 10.1111/1753-0407.12024

[bib33] de Boer IH, Tinker LF, Connelly S et al. Calcium plus vitamin D supplementation and the risk of incident diabetes in the Women's Health Initiative. Diabetes Care 2008; 31: 701–707. 18235052 10.2337/dc07-1829 PMC3046029

[bib34] Jorde R, Sollid ST, Svartberg J et al. Vitamin D 20 000 IU per week for five years does not prevent progression from prediabetes to diabetes. J Clin Endocrinol Metab 2016; 101: 1647–1655. 26829443 10.1210/jc.2015-4013

[bib35] Vasikaran S, Eastell R, Bruyère O et al. Markers of bone turnover for the prediction of fracture risk and monitoring of osteoporosis treatment: a need for international reference standards. Osteoporos Int 2011; 22: 391–420. 21184054 10.1007/s00198-010-1501-1

[bib36] Garnero P. The contribution of collagen crosslinks to bone strength. BoneKEy Rep 2012; 1: 182. 24363926 10.1038/bonekey.2012.182 PMC3868729

[bib37] Poundarik AA, Wu PC, Evis Z et al. A direct role of collagen glycation in bone fracture. J Mech Behav Biomed Mater 2015; 52: 120–130. 26530231 10.1016/j.jmbbm.2015.08.012 PMC4651854

[bib38] Yamagishi S, Nakamura N, Suematsu M et al. Advanced glycation end products: a molecular target for vascular complications in diabetes. Mol Med 2015; 21 (Suppl 1): S32–S40. 26605646 10.2119/molmed.2015.00067 PMC4661053

[bib39] Valcourt U, Merle B, Gineyts E et al. Non-enzymatic glycation of bone collagen modifies osteoclastic activity and differentiation. J Biol Chem 2007; 282: 5691–5703. 17142454 10.1074/jbc.M610536200

[bib40] Saito M, Fujii K, Mori Y et al. Role of collagen enzymatic and glycation induced cross-links as a determinant of bone quality in spontaneously diabetic WBN/Kob rats. Osteoporos Int 2006; 17: 1514–1523. 16770520 10.1007/s00198-006-0155-5

[bib41] Karim L, Bouxsein ML. Effect of type 2 diabetes-related non-enzymatic glycation on bone biomechanical properties. Bone 2016; 82: 21–27. 26211993 10.1016/j.bone.2015.07.028 PMC4679472

[bib42] Bi Y, Wang W, Xu M et al. Diabetes genetic risk score modifies effect of bisphenol a exposure on deterioration in glucose metabolism. J Clin Endocrinol Metab 2016; 101: 143–150. 26523527 10.1210/jc.2015-3039

[bib43] Takizawa M, Suzuki K, Matsubayashi T et al. Increased bone resorption may play a crucial role in the occurrence of osteopenia in patients with type 2 diabetes: Possible involvement of accelerated polyol pathway in its pathogenesis. Diabetes Res Clin Pract 2008; 82: 119–126. 18774197 10.1016/j.diabres.2008.07.008

[bib44] Vasikaran S, Cooper C, Eastell R et al. International Osteoporosis Foundation and International Federation of Clinical Chemistry and Laboratory Medicine position on bone marker standards in osteoporosis. Clin Chem Lab Med 2011; 49: 1271–1274. 21605012 10.1515/CCLM.2011.602

[bib45] Wu ZQ, Zhang Y, Xie E et al. High uric acid (UA) negatively affects serum tartrate-resistant acid phosphatase 5b (TRACP 5b) Immunoassay. PloS One 2016; 11: e0147554. 26800211 10.1371/journal.pone.0147554 PMC4723035

[bib46] Durup D, Jorgensen HL, Christensen J et al. A reverse J-shaped association of all-cause mortality with serum 25-hydroxyvitamin D in general practice: the CopD study. J Clin Endocrinol Metab 2012; 97: 2644–2652. 22573406 10.1210/jc.2012-1176

[bib47] Yeap BB, Chubb SA, Flicker L et al. Associations of total osteocalcin with all-cause and cardiovascular mortality in older men. The Health In Men Study. Osteoporos Int 2012; 23: 599–606. 21359669 10.1007/s00198-011-1586-1

[bib48] Currie CJ, Peters JR, Tynan A et al. Survival as a function of HbA(1c) in people with type 2 diabetes: a retrospective cohort study. Lancet 2010; 375: 481–489. 20110121 10.1016/S0140-6736(09)61969-3

